# The PET and LIM1-2 domains of testin contribute to intramolecular and homodimeric interactions

**DOI:** 10.1371/journal.pone.0177879

**Published:** 2017-05-18

**Authors:** Stefano Sala, Marie Catillon, Ermin Hadzic, Elisabeth Schaffner-Reckinger, Marleen Van Troys, Christophe Ampe

**Affiliations:** 1 Department of Biochemistry, Ghent University, Ghent, Belgium; 2 Cytoskeleton and Cell Plasticity Lab, Life Sciences Research Unit - FSTC, University of Luxembourg, Luxembourg, Luxembourg; Hungarian Academy of Sciences, HUNGARY

## Abstract

The focal adhesion protein testin is a modular scaffold and tumour suppressor that consists of an N-terminal cysteine rich (CR) domain, a PET domain of unknown function and three C-terminal LIM domains. Testin has been proposed to have an open and a closed conformation based on the observation that its N-terminal half and C-terminal half directly interact. Here we extend the testin conformational model by demonstrating that testin can also form an antiparallel homodimer. In support of this extended model we determined that the testin region (amino acids 52–233) harbouring the PET domain interacts with the C-terminal LIM1-2 domains *in vitro* and in cells, and assign a critical role to tyrosine 288 in this interaction.

## Introduction

**Fig 1:**
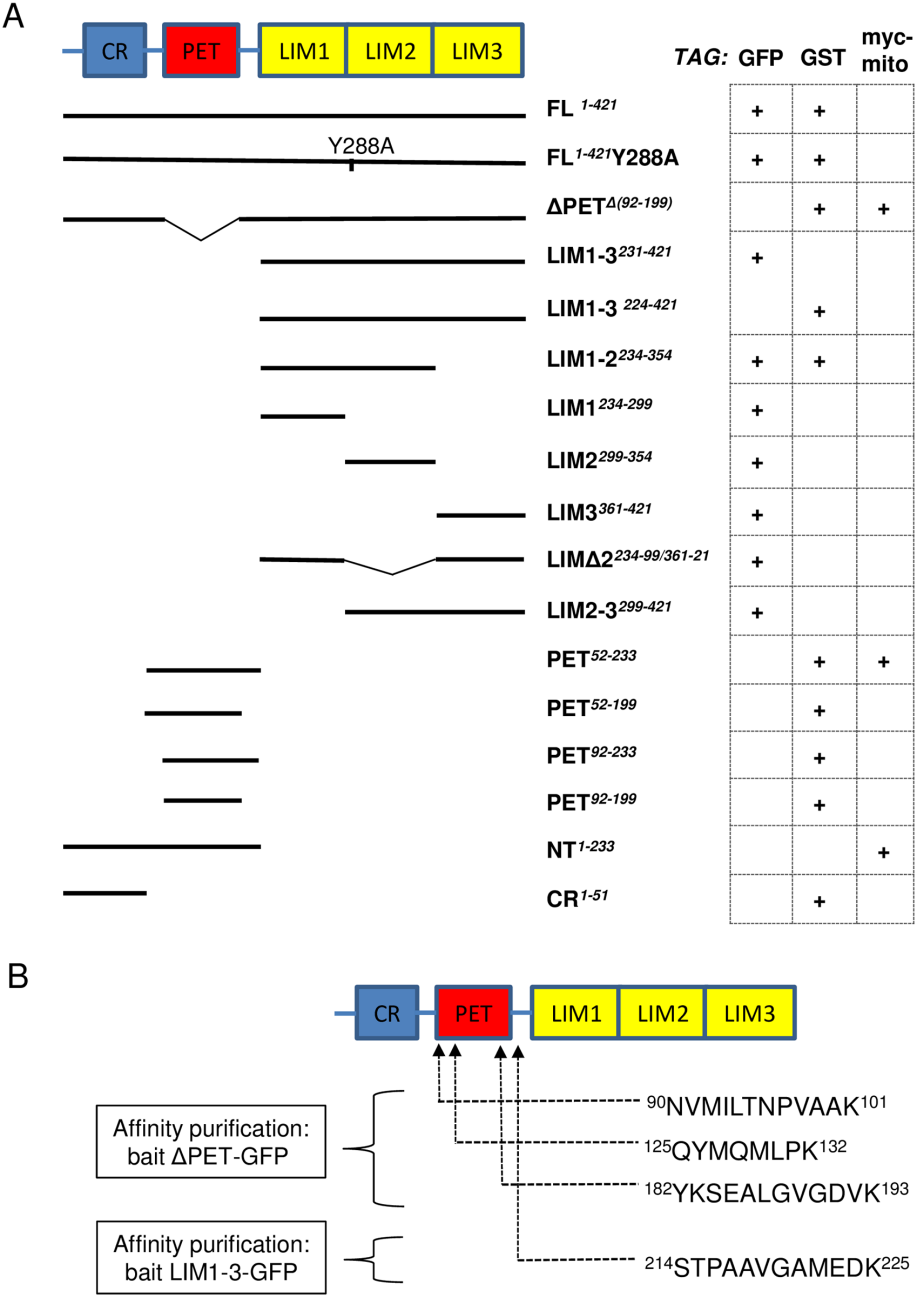
Tagged testin constructs. A) Scheme of the structural domains of testin and of the different modular testin variants used in this study. Testin variants are coupled to GFP, GST or myc/mito (see Methods for details, ‘+’ indicates that a particular tagged version of the testin variant is used in one or more of the experiments presented in the manuscript). CR: cysteine rich; PET: Prickle, Espinas, Testin; LIM: Linl-1, Isl-1, Mec-3, NT: N-terminal part. Numbers indicate start and end of protein variants based on the numbering of full length (FL) human testin isoform 1 (Database ID: NP_056456.1). B) Sequence and location of testin peptides originating from endogenous testin that were identified using a mass spectrometry-based approach in the tryptic digests of complexes affinity purified using a GFP-nanobody from lysates of HeLa cells expressing ΔPET-GFP or LIM1-3-GFP as bait (PRIDE dataset identifier: PXD005058) [18,20].

Human testin (NP_056456.1), encoded by the *TES* gene (Unigene Hs.592286), is a modular scaffold protein consisting of a cysteine rich (CR) region, a central PET (Prickle, Espinas, Testin) domain and three LIM (Linl-1, Isl-1, Mec-3) domains together constituting the C-terminal half of testin (Fig 1A) [1–3]. LIM domains contain two zinc-fingers and are known to be scaffolds for protein-protein interactions [4]. The interest in testin mainly arises from the fact that it has been shown to be downregulated in an increasing number of human tumour types where its downregulation correlates with disease progression [5–10]. In multiple studies in mice and tumour cells, overexpression of testin leads to increased cell spreading and apoptosis, inhibition of tumour cell proliferation and migration, or a reduction of tumour formation in nude mice [1,9,11–13]. In addition, *Tes* knockout mice have an enhanced susceptibility to develop drug-induced gastric carcinomas [14].

Although a role for testin in p38-signalling has been suggested [9], previous reports mainly provide evidence that testin is a component of the actin cytoskeleton. It interacts with multiple actin cytoskeletal proteins (zyxin, α-actinin, mammalian enabled (MENA), vasodilator stimulated phosphoprotein (VASP), ena/VASP-like protein (Evl), spectrin, paxillin, talin and transforming growth factor beta-1-induced transcript 1 (Hic-5) and is present in actin-rich structures such as stress fibres, integrin based-cell-substrate contacts (focal adhesions) and cadherin-based cell-cell contacts (adherens junctions) [1,2,15–18]. We recently reported a proteomics-based interactome study using the different testin domains as bait, detailing more than 100 proteins newly identified as present in testin-containing co-complexes in a domain-dependent manner [18]. This is consistent with the intriguing observation that the subcellular localization of testin is different for the full length protein than for engineered parts of the protein. Indeed, the N-terminal half of testin and the C-terminal half, differentially localize to stress fibres and focal adhesions, respectively, whereas the full length protein (FL) displays a diffuse localization in the cytosol with only a small fraction enriched in actin-rich structures [2,3,18,19]. To explain this, a conformational model was formulated a decade ago: testin is proposed to be present in a closed and an open conformation [1]. The open testin form is suggested to allow e.g. LIM-domain based recruitment to focal adhesions and interaction with known focal adhesion components such as zyxin.

During the interactomics study of testin [18], we unexpectedly found that ectopically expressed testin-variants and endogenous testin reside in one complex raising the possibility that testin forms dimers. In the present study, we used purified recombinant testin to show that it effectively exists in a monomeric and dimeric form. In addition, using experiments *in vitro* and in cells, we show that the region 52–233 containing the PET domain directly interacts with the LIM1-2 domains and contributes to the inter- or intramolecular interaction. This assigns a function to the PET domain and its flanking regions as an additional protein interaction module within testin. Because testin is capable of forming a monomer or a dimer, we conclude that the conformational repertoire of testin is more extensive than the open/closed monomeric transition previously described [1].

## Results

### Full length testin forms a dimer

In an extensive testin interactome study in HeLa cells we used a set of green fluorescent protein (GFP)-fused testin protein variants as bait to analyse, via affinity purification-mass spectrometry (AP-MS), the composition of the protein complexes these testin variants participate in. This is extensively reported in [18]. Of interest here, testin peptides that could only originate from endogenous testin, were present in the tryptic digest of the co-precipitated complexes using the ΔPET-GFP and the LIM1-3-GFP variant (Fig 1A) as bait. Indeed, as indicated in Fig 1B, we identified three PET domain peptides in a complex with the testin variant lacking the PET domain (ΔPET-GFP) and one peptide of the linker region between the PET and LIM1 domains in association with LIM1-3-GFP. In this AP-MS assay, recovery of testin peptides from endogenous protein could either result from an indirect recruitment (i.e. via VASP or zyxin or another unknown partner) or from a direct testin-testin intermolecular interaction. We explored the latter possibility.

**Fig 2:**
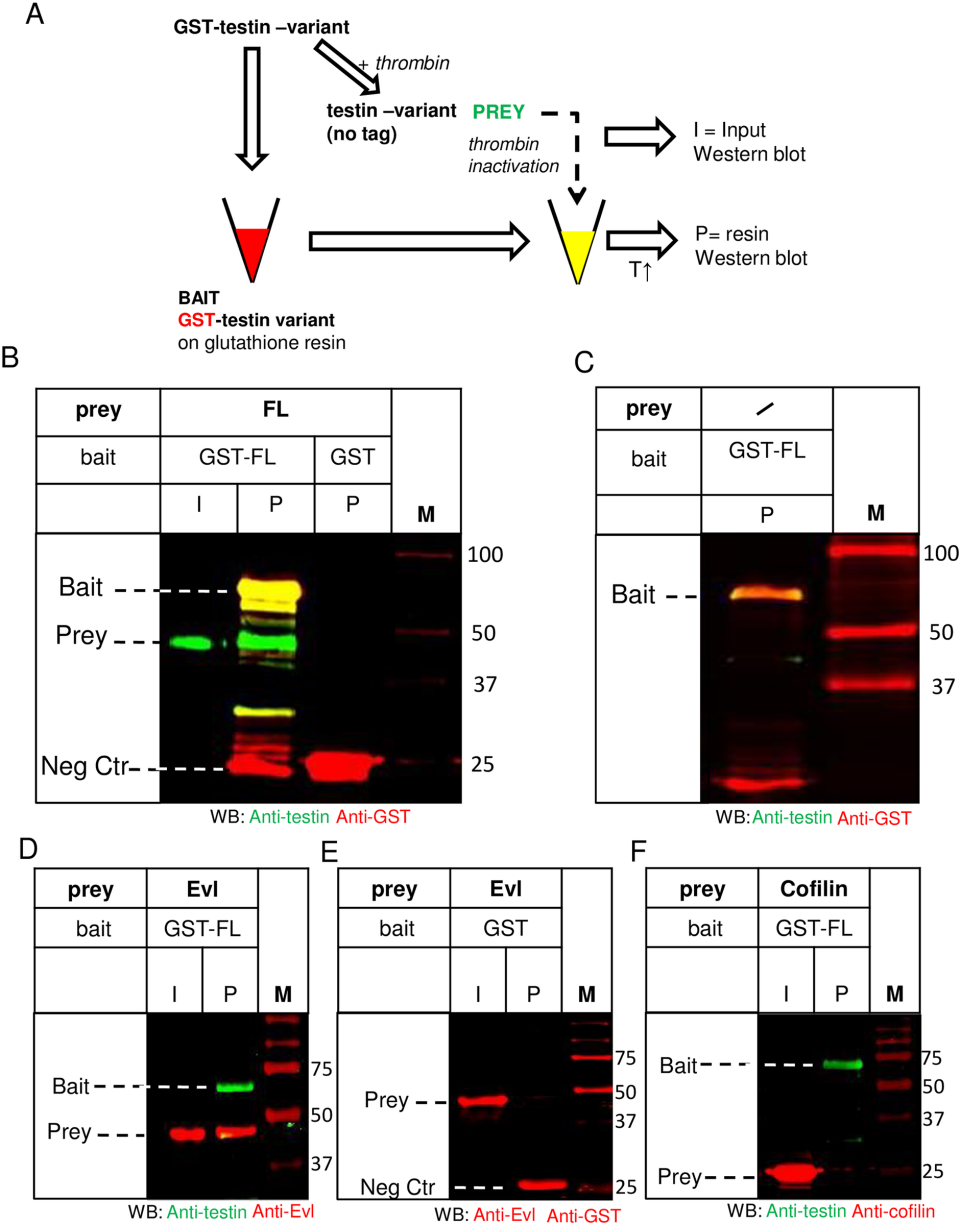
Full length testin interacts with full length testin in vitro. A) General Scheme of affinity purification used to produce data in several figures, to demonstrate an interaction of testin with other testin variants. In panel B of this figure, recombinant GST-FL was either used as bait on glutathione-sepharose or in parallel treated with thrombin to remove GST to be used as prey in an untagged form. Thrombin was inactivated prior to addition of this soluble form (input: I) to the resin with GST bound protein. After washing the resin, bound proteins were eluted with heated sample buffer and thus contain both bait and potential prey proteins (affinity purified: P). Proteins were detected either by Western Blotting (Figs 2B–2F and 4D) or Coomassie (Figs 4A–4C and 5A–5D), T = temperature. B) Immobilised recombinant GST-FL (bait) was incubated with soluble recombinant untagged FL (prey). A Western blot using anti-testin (green) and anti-GST (red) antibodies of the input (I) and proteins on the resin (P) is shown. FL prey (approx. 50 kDa) was present on the resin together with the bait GST-FL (lane ‘GST-FL/P’). Input (I) shows the untagged FL in solution. Recombinant immobilised GST was used as a negative control (lane ‘GST/P’). C) Western blot analysis (anti-testin (green), anti-GST (red)) of a mock buffer control incubated with immobilised GST-FL on resin. Similar as in B, the buffer contains inactivated thrombin but no soluble FL prey. Untagged FL is absent on the resin (P) (compare to Fig 2B, lane ‘GST-FL/P’) indicating that possible residual thrombin activity is not cleaving the GST-FL on the resin. D, E, F) Immobilised recombinant GST-FL (bait) was incubated with soluble untagged Evl (positive control, D) or cofilin (negative control, F) as preys. Immobilised GST was incubated with Evl (prey) and used as additional negative control (E). Western blot analysis of inputs (I) and proteins on the resin (P) is shown using anti testin (green), anti-Evl, anti-cofilin and anti-GST (red) antibodies. Untagged Evl (prey) is present on the GST-FL resin (lane P, 2D). Positions of bait, prey and negative control bait (Neg Ctr) are indicated in each panel. M: marker proteins (kDa).

We first used an *in vitro* binding assay with purified components to determine whether full length (FL) testin directly interacts with itself (scheme Fig 2A). We immobilized recombinant glutathione-S-transferase GST-FL (Fig 1A), produced in *E*. *coli*, on glutathione–coupled sepharose as bait and incubated this resin with non-tagged recombinant FL as prey (input (I) in Fig 2B). The latter was obtained from GST-FL by exploiting a thrombin recognition site present between the GST-tag and FL, followed by inactivation of thrombin (see Methods). As negative control, we used GST coupled resin that was similarly incubated with soluble untagged FL. The interaction between immobilized GST-FL and soluble FL was monitored by western blot analysis of the proteins on the resin using anti-testin and anti-GST antibodies. Fig 2B shows that FL binds to the GST-FL on the resin (lane ‘GST-FL/P’) but not to the GST resin (lane ‘GST/P’), indicating a direct testin-testin interaction. We also mock-incubated the FL-GST-beads with buffer lacking soluble FL as prey but containing a similar amount of thrombin that was inactivated in an identical manner. Under these conditions, untagged FL is not present in the analysed resin (Fig 2C, lane ‘P’) and this excludes that the FL, identified as bound to the GST-FL resin in Fig 2B (lane ‘GST-FL/P’), was a (residual thrombin) breakdown product of GST-FL on the resin. We also incorporated an additional positive and negative control in the experimental setup (Fig 2D–2F). The GST-FL coupled resin retained the known testin partner Evl (Fig 2D, positive control) but not cofilin (Fig 2F, negative control). Together these data demonstrate that purified FL is capable of forming a direct intermolecular interaction *in vitro*.

**Fig 3:**
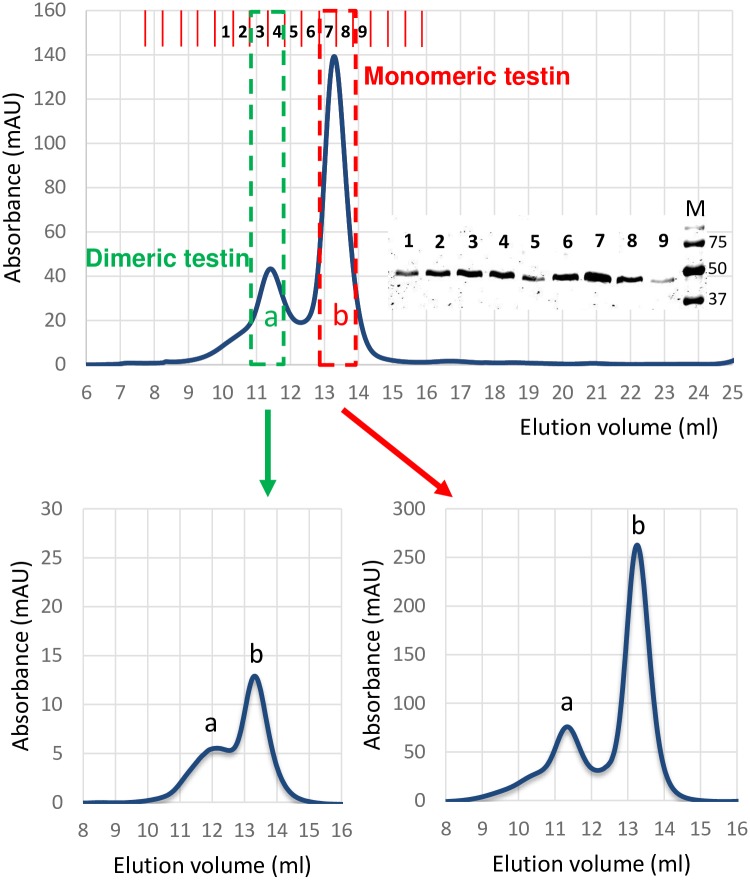
Size exclusion chromatography reveals dimer formation of testin *in vitro*. Top panel: Chromatogram of SEC of purified recombinant FL. Absorbance (280 nm) is plotted versus elution volume (ml). Two elution peaks are present (labelled a and b). Bold numbers 1–9 on top of chromatogram indicate the collected fractions which all contain testin as visualized by Coomassie staining after SDS-PAGE (see S1A Fig for column calibration). Bottom panels: Re-analysis by SEC of pooled fractions 3 and 4 (bottom left) or 7 and 8 (bottom right) of the primary SEC (boxed in top panel). Fractions 7 and 8 were first pooled and concentrated to a final concentration of 3,1 mg/ml prior to re-analysis with SEC. In both secondary runs two peaks eluted corresponding to dimeric (a) and monomeric (b) testin evidencing that both forms are in dynamic equilibrium with each other.

This predicts that testin is capable of forming homomeric species. To demonstrate this, we performed size exclusion chromatography (SEC) with recombinant untagged FL testin using a calibrated column. A typical separation pattern and the analysis of selected fractions using SDS-PAGE and Coomassie staining is shown in Fig 3 (top panel). Two testin containing peaks (labelled a and b) eluted from the column. Based on the elution volumes measured at the maximum of the peaks and a molecular weight (MW) calibration curve (S1A Fig), we estimated the MW of the two corresponding testin species. For proteins eluting in peak b (approx. elution volume 13 ml), we derive a MW of ~60 kDa which is slightly higher than the calculated MW of a testin monomer (47996 Da) (see Discussion). Similarly, we derive for peak a (eluting at approx. 11 ml) a MW of ~ 98 kDa and this is close to the calculated MW of 95992 Da for a dimeric testin form. To exclude that the presence of a dimer peak is due to oxidation of the sample, we treated fractions 3 and 4 with sample buffer lacking the reducing agent dithiothreitol followed by Coomassie analysis (S1B Fig). A monomeric testin signal (~50 kDa) was detected and no higher molecular weight signal resulting from an oxidation of the protein was visible. These results are therefore most consistent with the presence of a monomeric and a dimeric testin population. Fractions 3 and 4 representing the main part of the dimer peak (a) were pooled and subjected to a second SEC on the same column. Fig 3 (bottom left) shows this results in the formation of a new monomer population in the time in between the two SEC runs. In addition, we pooled the tail fractions 7 and 8 of the monomer peak (b) from the first SEC, concentrated and reanalysed them by SEC. The chromatogram in Fig 3 (bottom right) shows the presence of a new dimer population formed from the original monomeric pool. Together this demonstrates a dynamic equilibrium between the monomer and dimer population. Taking the results from the SEC experiments together with the binding data, we demonstrate that testin is capable of dynamically forming a homodimer *in vitro*.

### The PET containing region of testin directly interacts with LIM1-2 domains in vitro

Garvalov *et al*. [1] showed that the N-terminal half of testin (containing the CR and PET domains) and the C-terminal half (containing the three LIM domains) directly interact. This was interpreted in terms of an intramolecular interaction, however the parts of the molecule mediating this interaction could also be at play in the dimer formation which we showed in the previous section. Therefore, we set out to more finely delineate which regions in testin are required for interacting with each other as this is relevant for dimer formation in addition to the possible intramolecular interactions. Additionally, it may allow defining whether the dimer is formed in a parallel or antiparallel manner.

**Fig 4:**
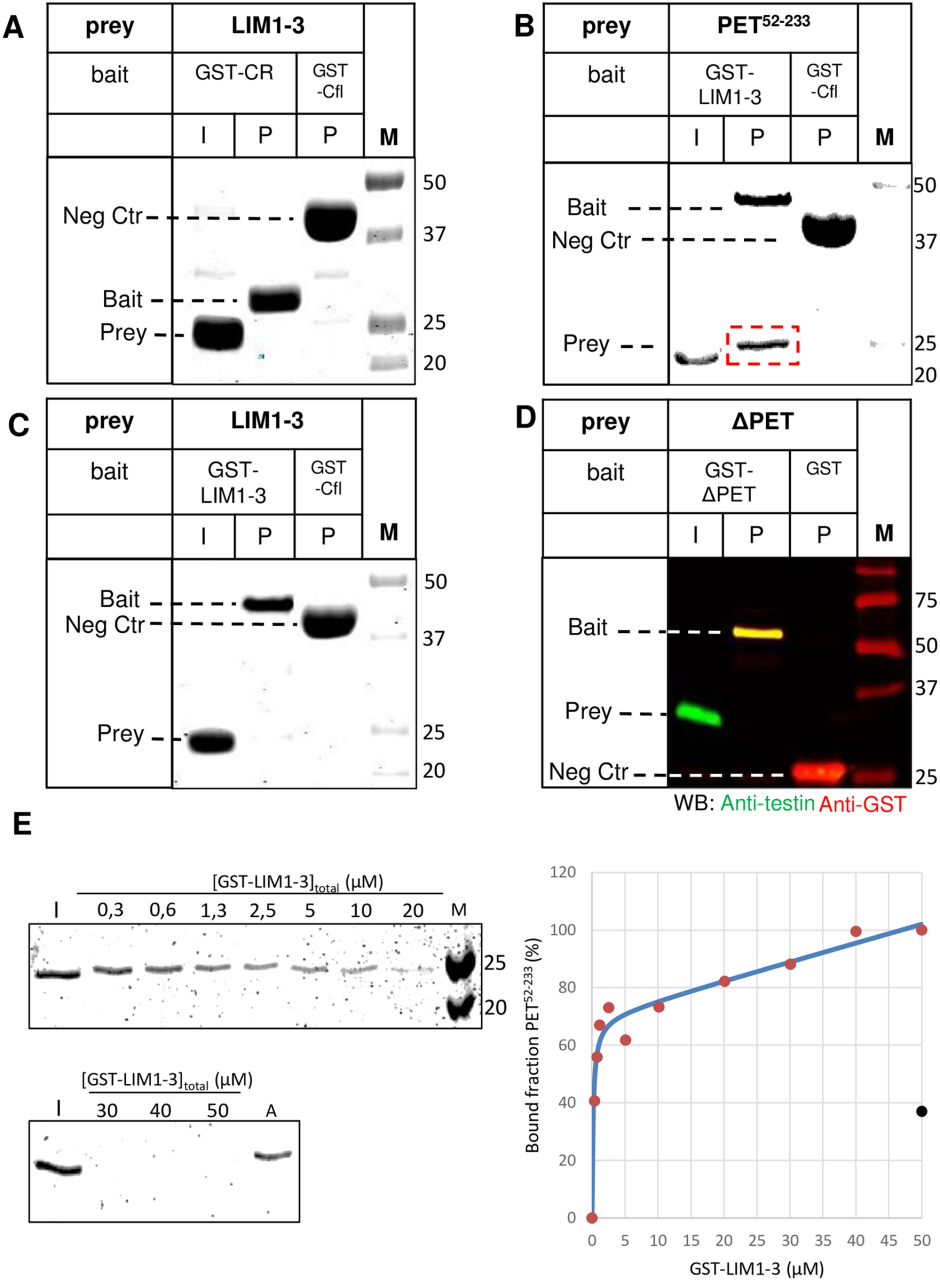
PET^52-233^ of testin directly interacts with LIM1-3 domains *in vitro*. A-D) The experimental setup is similar as shown in Fig 2A. A recombinant GST-testin variant used as bait (GST-CR (A), GST-LIM1-3 (B, C) or GST-ΔPET (D)) was trapped on glutathione resin and presented in immobilised form to a second untagged testin-variant in solution used as prey (LIM1-3 (A, C), PET^52-233^ (B) or ΔPET (D)). Coomassie stained SDS-PAGE analysis (A-C) or western blot analysis (D) using anti-testin (green) and anti-GST (red) antibodies is shown. Input (I) shows the untagged prey protein prior to incubation with the resin. Lanes indicated with P show the proteins present on the resin (immobilized bait and/or bound prey) after the incubation. GST resin (D) or GST-cofilin resin (A-C) incubated with the same untagged testin variant as prey were used as negative controls. Untagged protein bound to the GST-testin variant immobilised on the resin is highlighted by a red box (B). Positions of bait, prey and negative control (Neg Ctr) bait are indicated in each panel, M: marker proteins (kDa). E) The indicated concentrations of GST-LIM1-3 immobilized on glutathione-sepharose beads were prepared and incubated with 2.5μM PET^52-233^. For each LIM1-3 concentration (indicated as [GST-LIM1-3]_total_ (μM)), the level of unbound PET^52-233^ was analysed by Coomassie staining after SDS-PAGE (left). Aspecific binding was assessed by incubation of glutathione-sepharose beads, lacking LIM1-3, with a similar concentration of PET^52-233^ I: input, representing 2.5 μM PET^52-233^ in solution without incubation to LIM-1-3 coupled glutathione beads (reference for unbound 100% or bound 0%), A: aspecific binding of the PET^52-233^ ligand to beads without LIM1-3, M: molecular weight marker (kDa). (right) The % amounts of bound PET^52-233^ calculated from the measured intensities of the unbound material on gel (left) were plotted versus GST-LIM1-3 concentrations (graph right, red dots). The amount of aspecifically bound PET^52-233^ is represented by a black dot. The solid blue line in the graph is the fitted curve taking into account aspecific binding (see Materials and methods for details).

We applied an affinity pull down approach similar to the one shown in the scheme in Fig 2A, again using *E*. *coli* produced fragments of testin. One testin variant as bait was immobilised on the resin via its GST-tag and incubated with a purified untagged testin variant as prey in solution. Starting from the observation that the three C-terminal LIM domains interact with the N-terminal half (Garvalov *et al*., [1]), we first assessed which region of the N-terminal half of testin is responsible for the interaction with LIM1-3: CR or the region including the PET domain (residues 92–199, www.ebi.ac.uk/interpro/entry/IPR033724), here named PET^52-233^ (see Fig 1A). We tested the potential interactions of recombinant immobilised GST-CR with LIM-1-3 or, alternatively, GST-LIM-1-3 with PET^52-233^ (Fig 4A and 4B respectively, see Fig 1A for used variants). We used GST-cofilin resin as a negative control. After elution and SDS-PAGE the potential interactions were monitored by Coomassie staining. Fig 4A illustrates that there is no direct interaction between LIM-1-3 and CR. In contrast, Fig 4B shows a direct interaction between PET^52-233^ and the immobilised LIM1-3 domains (red box, Fig 4B). These *in vitro* data demonstrate that the PET^52-233^ region possesses the interaction information within NT. In a similar way, we assessed whether LIM1-3 could be retained on a GST-LIM1-3 resin (Fig 4C), which was not the case, illustrating that the observed signal in Fig 4B originates from PET^52-233^ and not from breakdown of the GST-LIM1-3 on the resin. To additionally validate the role of the region harbouring the PET domain for an intra- and/or intermolecular testin interaction, we used a testin variant lacking the PET domain (deletion of residues 92–199). Fig 4D shows that ΔPET (prey) and immobilised GST-ΔPET (bait) indeed do not interact despite the presence of the LIM domains.

We performed a supernatans depletion assay [21] to determine the affinity of the PET^52-233^ LIM1-3 interaction. We incubated different concentrations of GST-LIM1-3, coupled to glutathione-sepharose beads, with 2.5 μM of PET^52-233^ and measured its depletion from the supernatans on Coomassie stained gels (Fig 4E). From the intensities on gel, we calculated the bound PET^52-233^ fractions and plotted these versus the LIM1-3 concentrations. Beads lacking GST-LIM1-3 were similarly incubated with 2.5 μM of PET^52-233^ to assess aspecific binding. This revealed that measurable non-specific binding of PET^52-233^ to the beads occurred. We therefore took this in account in the model for fitting (see Materials and methods section for details). This allowed determining a dissociation equilibrium constant (*K*_d_) for the interaction between isolated PET^52-233^ and LIM1-3 domains which is within the nanomolar range (*K*_d_ = 0,18 μM ± 0,06).

**Fig 5:**
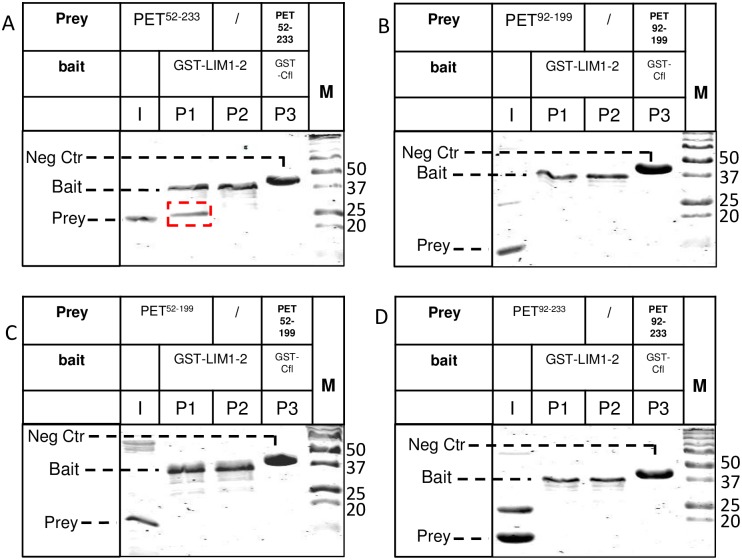
The PET domain of testin is not sufficient for interaction with LIM1-2 domains *in vitro*. The experimental setup is similar as the scheme in Fig 2A. Immobilised GST-LIM1-2 on glutathione resin was incubated with untagged PET^52-233^(A), PET^92-199^(B), PET^52-199^ (C) or PET^92-233^ (D) in solution, used as preys. Coomassie stained SDS-PAGE analysis is shown: input (I) shows the untagged prey protein prior to incubation with the resin. Lanes indicated with P1 show the proteins present on the resin. We here included an extra negative control: the immobilised baits on the resin were mock-incubated with a solution lacking the soluble preys as in some cases the bait construct is prone to degradation during immobilization on the resin (lanes labelled P2). GST-cofilin resin was used as second negative control in each setup (lanes P3). Untagged prey protein PET^52-233^ bound to the GST- LIM1-2 testin variant immobilised on the resin is highlighted by a red box (A). Positions of bait, prey and negative control (Neg Ctr) bait are indicated in each panel, M: marker proteins (kDa).

We further tried narrowing down the interaction sites. A GST-testin variant containing only LIM domains 1 and 2, used as bait, is sufficient for the interaction with PET^52-233^ (Figs 1A and 5A). Additional experiments in HeLa cells with LIM2-3, LIMΔ2 (a testin variant containing LIM domain 1 fused to 3, Fig 1A) as well as the individual LIM1 and LIM2 domains provide no evidence for interaction with PET^52-233^ suggesting that both the first and second LIM domains are necessary to ensure this interaction (S4B–S4E Fig and Discussion). In addition to PET^52-233^, we constructed three truncated variants covering only the PET domain (PET^92-199^) or the PET domain flanked by one of the two linker regions (PET^52-199^ and PET^92-233^) (Fig 1A). These truncated PET variants as well as the PET^52-233^ variant were used as prey to assess potential interactions with the immobilized LIM1-2 baits. Unlike PET^52-233^ which interacted with the LIM1-2 bait (lane P1 in Fig 5A), neither of the three truncated PET variants was retained by the LIM 1–2 bait (lanes labelled P1 in Fig 5B–5D) demonstrating that the PET^92-199^ domain and its flanking linker regions are necessary to ensure a detectable interaction with the LIM domains under the tested conditions. In a similar way, we found that the three truncated PET variants were not retained by a LIM1-3 bait excluding that a weak contribution to the interaction was due to the third LIM domain (S2B–S2D Fig). This lack of interaction between the isolated PET^92-199^ domain and LIM1-2 could be caused by loss of its folded conformation upon eliminating the flanking regions of the PET domain. We therefore performed circular dichroism (CD) measurements on both variants (S3 Fig). Although the spectra are different they reveal that both PET^52-233^ and PET^92-199^ are structured and likely dominated by α-helices (consistent with findings for the Drosophila prickle PET domain, [22]). Thus, unfolding of the PET domain cannot be the cause of its lack of binding to LIM1-2 in the context of 92–199, 52–199 or 92–233.

Taken together, these data establish that the PET domain and its flanking regions interact with nanomolar affinity with the LIM1-2 domains and strongly suggests that, using this interaction, testin adopts either a monomeric conformation or exists as an antiparallel homodimer.

### The region 52–233 containing the PET domain interacts with the testin LIM1-2 domains in a cellular context

**Fig 6:**
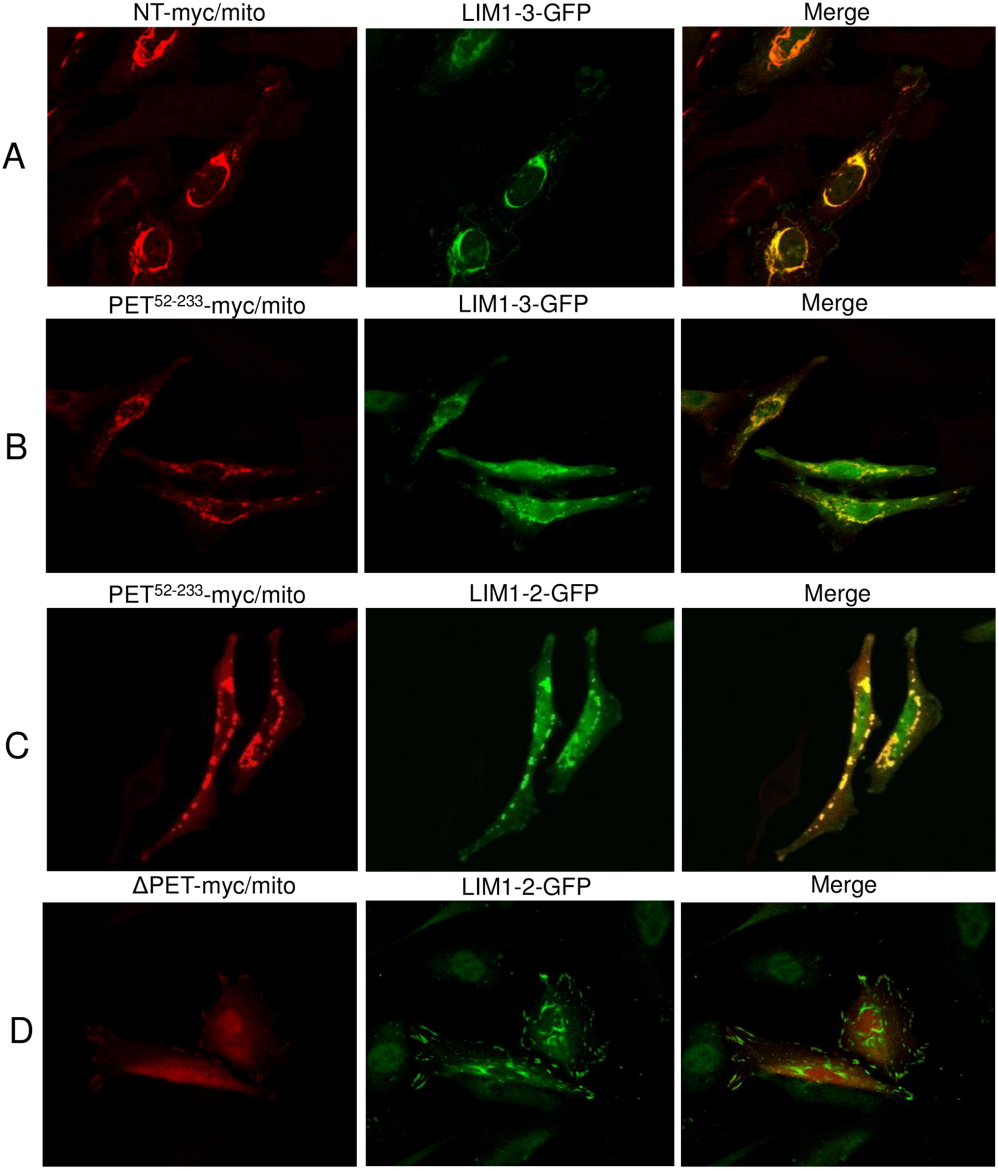
The region 52–233 containing the PET domain of testin directly interacts with LIM1-2 domains in cells. Immunofluorescence staining of myc/mito-coupled (bait) and GFP-coupled (prey) testin constructs in HeLa cells. Myc signal (red), GFP signal (green) and merge are shown for each condition. Colocalisation is observed for NT-myc/mito and LIM1-3-GFP (A), PET^52-233^-myc/mito and LIM1-3-GFP (B) and PET^52-233^-myc/mito and LIM1-2-GFP (C). Absence of colocalisation is observed for ΔPET^Δ92-199^-myc/mito and LIM1-2-GFP (D). See also S4 Fig.

We set out to validate the interaction between PET^52-233^ and LIM1-2 in HeLa cells using an *in cellulo* recruitment assay with myc/mito tagged testin variants. The mito tag was originally identified as the mitochondrial targeting domain of the ActA proteins of the bacteria *Listeria monocytogenesis* [23], and subsequently successfully used to artificially target the mito-tagged fusion proteins together with their interaction partners in cells to the mitochondrial surface [16,24]. The expressed myc-mito tagged testin variants served as intracellular baits for ectopic recruitment of preys. The myc-tag was used for bait detection. A GFP tagged testin variant is co-transfected as prey and co-localisation of the latter at the mitochondria with the mito tagged bait is indicative of an interaction of both variants in a cellular context. Fig 6A demonstrates that, in HeLa cells, LIM1-3-GFP (Fig 1A) colocalizes with NT-myc/mito. Similarly, LIM1-3-GFP was also recruited by PET^52-233^-myc/mito (Fig 6B) as was LIM1-2-GFP (Fig 6C). As expected based on the *in vitro* data, ΔPET myc/mito, lacking the PET^92-199^ domain, did not recruit LIM-1-2-GFP (Fig 6D). The latter result also indicates it is unlikely that the recruitment of the LIM domains observed above was indirect via the scaffolding interaction of the LIM domains. This illustrates that the region 52–233 containing the PET domain and LIM1-2 are essential for an intermolecular testin interaction in a cellular context.

### Y288 is (structurally) important for the testin-testin interaction

**Fig 7:**
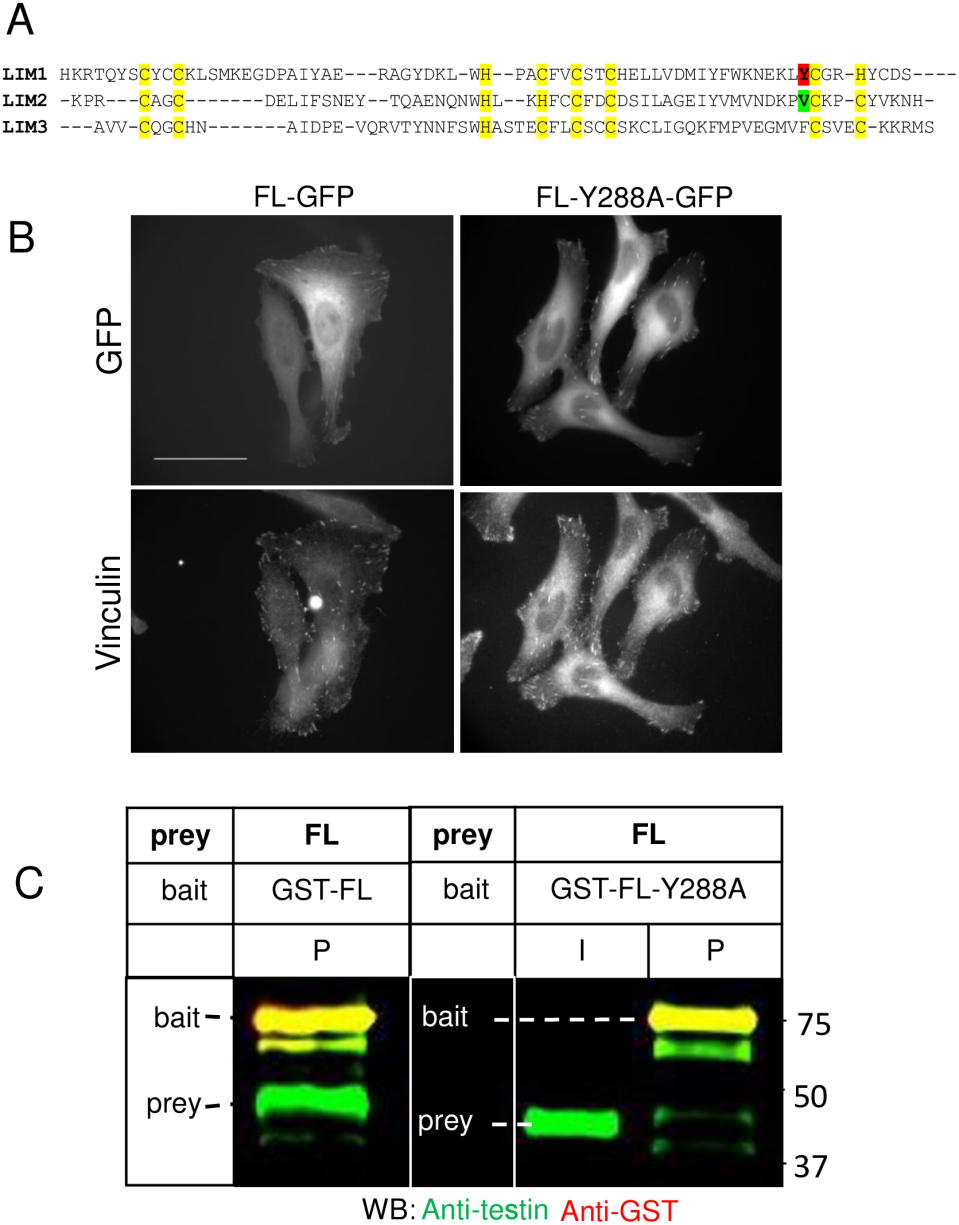
Y288 is important for testin-testin interaction. A) Multiple sequence alignment of the LIM domains of testin. Zinc coordinating cysteines (C) and histidines (H) are indicated with yellow highlight. Tyrosine (Y) 288 and valine (V) 348 are indicated in red and green respectively. This valine in LIM2 makes a hydrophobic stacking interaction with LIM3 based on the crystal structure in (Database id: 2xqn, [3]) B) Localization of GFP-coupled FL and FL-Y288A constructs in fixed HeLa cells. The GFP and vinculin signals are shown. FL and Y288A are distributed throughout the cytosol and present in focal adhesions. C) Immobilised recombinant GST -FL or GST-FL-Y288A (bait) was incubated with a similar amount of soluble recombinant untagged FL (prey) followed by western blot analysis of the protein on the resin using anti-testin (green) and anti-GST (red) antibodies. FL (approx. 50 kDa) is present on the resin (P) together with the immobilised GST-FL and immobilised GST-FL-Y288A. Input (I) shows the untagged FL prey in solution. Note the strongly reduced amount of bound FL when FL-Y288A mutant is used as bait. Positions of bait and prey are indicated in each panel. Molecular weights (kDa) are indicated.

The *in vitro* and *in cellulo* demonstrated interaction between PET^52-233^ and LIM1-2, may support conformational regulation of testin by allowing an intra- and/or intermolecular testin interaction in cells. The atomic 3D structure of the PET domain is still unknown, but the structure of LIM2-3 in complex with the partner proteins Mena and Arp7A (actin related protein 7A) has been derived [3] (Database id: 2xqn). Fig 7A shows the alignment of the three LIM domain sequences of testin demonstrating that LIM1 tyrosine 288 aligns to LIM2 valine 348. The latter residue makes an important stacking interaction between the LIM2 and LIM3 domains (derived from visual inspection of structure 2xqn [3]). We hypothesized that the LIM1 residue Y288 may have a similar role in a stacking interaction between LIM1 and LIM2 and thus that Y288 may determine the relative orientation of LIM1 and LIM2. We mutated Y288 to alanine in the full length testin context (Fig 1A). This substitution did not impair testin localization to focal adhesions in HeLa cells (Fig 7B). GST-FLY288A mutant or GST-FL on resin (baits) were, in parallel, incubated with equal amounts of FL (prey) to compare their capacity to form a dimer *in vitro*. Fig 7C demonstrates that both interactions take place (see ‘P’), but the affinity of FL for the mutant is strongly reduced. These data suggest that Y288 between the LIM1 and LIM2 domains is of structural importance for the interaction with PET^52-233^.

## Discussion

In this study, we demonstrated that the focal adhesion protein testin exists, in addition to its monomeric form, as an antiparallel (or head to tail) homodimer. This is not unprecedented for focal adhesion proteins since vinculin and talin are also capable of self-associating either into homodimeric protein complexes or (closed) monomeric species [25–27]. Similarly, the Ezrin/radixin/moesin (ERM) family members, proteins connecting the actin cytoskeleton to the plasma membrane, are also capable of forming closed monomers and/or anti-parallel dimers through a direct interaction between their N- and C-termini [28]. The dimeric and monomeric forms of purified testin were observed in equilibrium in sequential size exclusion chromatography experiments. The estimated molecular weight of 60 kDa of the monomeric form which we obtained by SEC analysis deviates from the predicted one (approximately 48 kDa). This higher estimation may indicate that this form has an elongated structure resulting in a higher apparent molecular volume than expected. A similar observation has been previously made for other LIM domain containing proteins [see e.g. 29]. For the dimer, the estimated MW (98 kDa) deviates less, which suggests that this conformational state is becoming more spherical reducing its molecular volume.

We determined an affinity in the nanomolar range between PET^52-233^ within the N-terminal half of testin and LIM1-3. We further demonstrated that LIM1-2 form the major interaction site in the C-terminal half. The PET domain was originally found as part of the *Drosophila* prickle and espinas proteins. These proteins also contain three C-terminal LIM domains and are shown to play an important role in the planar cell polarity process [22]. Hitherto the functionality of the PET domain of testin was little investigated. In an interactomics study we recently showed that the testing PET^52-233^ region is a protein-protein interaction module [18]. Membrane binding has been suggested for *Drosophila* Prickle PET [30] and, interestingly, in that paper the three LIM domains of Prickle increased the stability of the PET domain. This is also in agreement with the physical interaction between these domains observed here. Here we propose an additional role for PET^52-233^ in regulating the conformation of testin by interacting with the first two LIM domains, enabling either an intramolecular or intermolecular interaction, the latter resulting in dimer formation. Interestingly, the PET domain was not sufficient to mediate this interaction. This is evidenced by lack of direct interaction of the isolated PET domain (residues 92–199) with LIM1-2 (or LIM1-3) as well as by the failure of dimer formation of the ΔPET variants. This also assigns a role to the regions adjacent to the PET domain; they appear to contain binding information. Interestingly, part of these linker regions between either the cysteine rich region and the PET domain or the PET domain and the LIM domains are predicted as intrinsically disordered regions by PrDOS (http://prdos.hgc.jp/cgi-bin/top.cgi). Collectively this indicates that the sequence information within PET^52-233^ that are required for binding LIM1-2 do not constitute a simple linear recognition motif. Both LIM1 and LIM2 domains are required for high affinity interaction since in a cellular context the PET^52-233^ region does not interact with the separate LIM1 or LIM2 domains, nor with the third LIM domain (LIM3) or the last two LIM domains (LIM2-3) (S4B–S4F Fig). This disagrees with a previous finding that demonstrated that the N-terminal half of testin directly interacts with the third LIM domain and not with LIM1-2 *in vitro* [31]. Different purification conditions (native, this work; denaturing and renaturing from inclusion bodies [31]) may be a possible explanation for this discrepancy. Our data are confirmed in mammalian cells and a further proof that the LIM1-2 domains are involved comes from our observation that mutating the LIM1 residue Y288 strongly diminishes dimer formation. This mutant still localizes to focal adhesions in cells, supporting its functionality (Fig 7B). The observed lack of dimer formation for FL-Y288 suggests that Y288 is an interface residue between the PET and LIM1-2 modules. Alternatively, this raises the possibility that introducing the mutation alters the orientation of the involved LIM domains relative to each other. This and whether Y288 is involved in opening the testin molecule is, however subject to future investigations.

**Fig 8:**
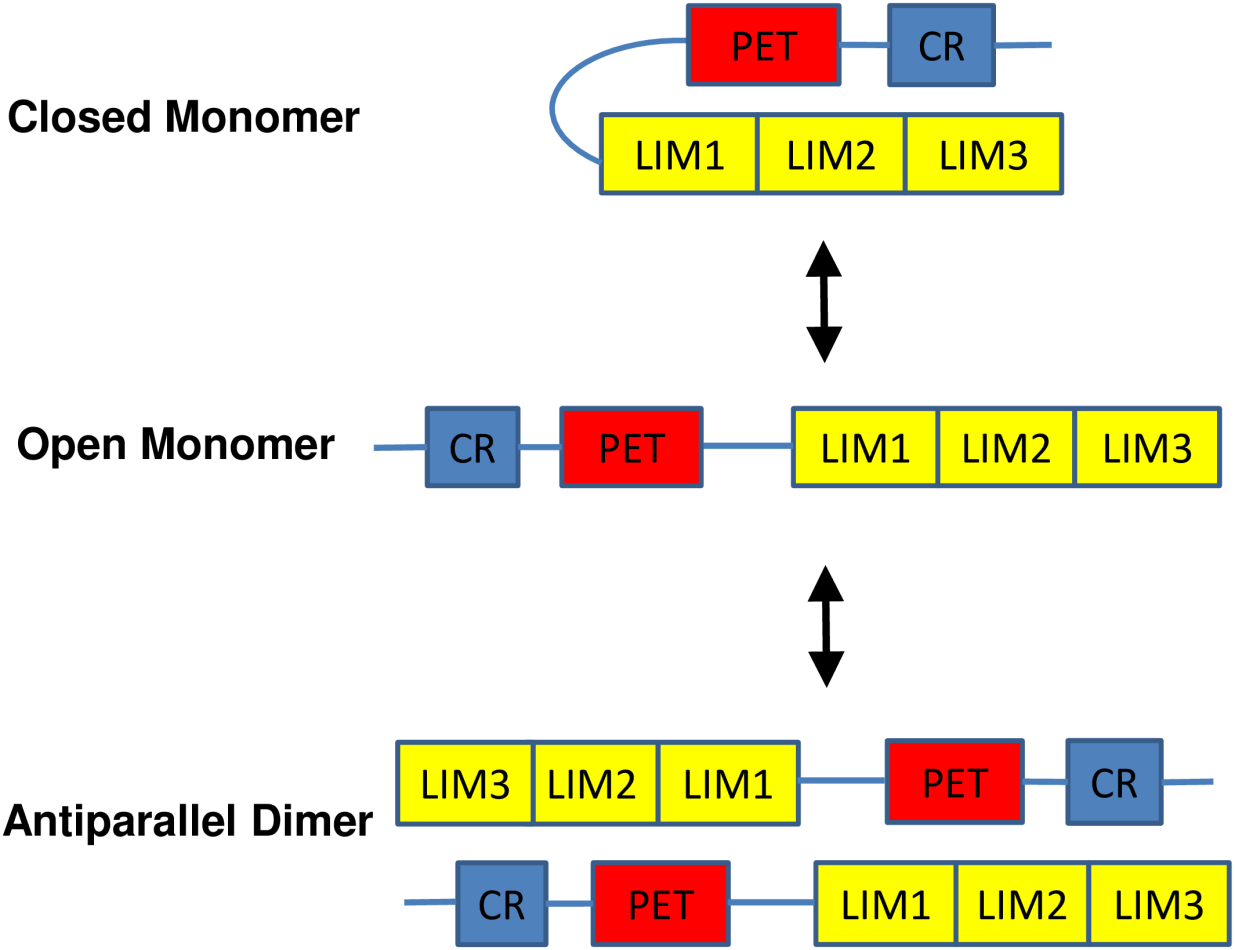
Conformational model of the testin protein. Testin can adopt an open active monomeric or a closed inactive monomeric conformation (as proposed by Garvalov *et al*. [1]) or an antiparallel dimeric conformation (this work). The activity status of the dimer is unknown (see Discussion). The interaction between PET^52-233^ and the LIM1-2 domains underlies formation of the dimer and/or closed monomer conformation.

Garvalov and coworkers have previously proposed that testin adopts an open or closed monomeric conformation, of which the latter can be formed by a direct interaction between its N-terminal and C-terminal halves[1]. This interaction however does not necessarily contradict the dimer formation we observed here. We accordingly extend the existing conformational model (Fig 8). Our finding that the PET^52-233^ region directly interacts with the C-terminal LIM1-2 domains and that this mediates the intra- and intermolecular testin interactions requires revising the previous conformational model of testin [1].

In Fig 8 we propose the existence of three and not two conformational states of the testin protein being a closed monomer, an open monomer and an antiparallel dimeric state. This may present another example of a 3D-domain-swapped system of a monomer and homodimer as observed for diphtheria toxin, and calcium and integrin binding protein [32,33]. At present, it is however unclear whether the dimer is an inactive form, similar to the closed monomer wherein specific domains or protein-protein interaction interfaces are inaccessible, or an active form. We indeed recently showed that the interactome of the full length protein is different from those of testin variants that only contain specific domains [18]. The mechanism that drives the conformational transitions between the closed monomer and the dimer in testin remains to be investigated but the open monomeric conformation could be a logical intermediate. In cells, these transitions are likely subject to regulation, which can involve either the PET- or the LIM1-2 domains or both. One scenario is that an interaction of testin with one of its partner proteins favours the (intermediate) open monomeric conformation ([1], thebiogrid.org/117572, [18]). Another attractive possibility is that the LIM1 domain becomes phosphorylated because this domain is a hotspot for phosphorylation events (Database ID: TES (human)). It is also important to note that, although most activities of testin that have been described so far are usually linked to testin at focal adhesions, testin has also been observed in the nucleolus [31] and in adherens junctions [17]. Testin may thus display additional specific cellular functionalities in which monomeric or homodimeric species have differential properties. The new model (Fig 8) may consequently form a more realistic basis to investigate the role of testin in cells, in particular in cancer cells.

## Materials and methods

### Testin encoding expression vectors

Testin variants (see Fig 1A) were expressed as recombinant GST-fusion proteins using pGEX-2TK (GE Healthcare) vectors in which the cDNAs encoding human FL (full length protein), PET^52-233^ (amino acids 52–233), PET^52-199^ (amino acids 52–199), PET^92-233^ (amino acids 92–233), PET^92-199^ (amino acids 92–199) or ΔPET (amino acids 1–91 fused to 200–421, i.e. deletion of PET domain) were cloned using the *Bam*HI and *Sma*I restriction sites in the multiple cloning site. For the GST-LIM1-3 construct, the FL-pGEX-2TK plasmid was digested with *Nco*I, eliminating the N-terminal CR and PET domains from the plasmid. The cDNAs encoding CR (CR domain) or FL-Y288A (Y288A substitution, FL here coupled to HIS tag) were cloned using the *Bam*HI and *Sal*I restriction sites in the multiple cloning site of pGEX-4T-2 (GE Healthcare). The cDNA encoding LIM1-2 (first 2 LIM domains, here coupled to a HIS tag) was cloned using *Eco*RI and *Sal*I restriction sites in pGEX-4T-2. All GST-fusion proteins carry a thrombin cleavage site allowing removal of the GST tag. The GST-cofilin construct has been described in [34]. For mammalian expression as eGFP-fusion proteins, FL-GFP was generated by cloning the cDNA corresponding to the full length protein (residues1–421) into the pEGFP-N3 vector (Clontech) [16]. For FL-Y288A, the construct was cloned into the pEGFP-N3 vector by DNA2.0. The cDNAs encoding LIM1-3, LIM1-2, LIM2-3, LIMΔ2, LIM1, LIM2 or LIM3 were cloned using the *Xho*I and *Bam*HI restriction sites in the multiple cloning site of pEGFP-C3 (Clontech) (Fig 1A). The cDNAs encoding NT or ΔPET were cloned using the *Sac*II and *Sph*I restriction sites, and the cDNA encoding PET^52-233^ was cloned using *Not*I and *Sal*I in the multiple cloning site of a pUHD10.3 vector encoding as tag a myc tag combined with the carboxy-terminal amino acids of ActA [23,24]. The latter is termed ‘mito tag’ as it allows targeting proteins to the mitochondrial surface [23]. All constructs were verified by sequencing.

### Cell culture and transfection

HeLa cells were cultured in Dulbecco’s Modified Eagle Medium with Glutamax supplemented with 10% fetal bovine serum and 1% penicillin/streptomycin (Gibco, Life Technologies) at 37°C and 5% CO_2_. Cells in a 6 well plate on coverslips (1.2x10^5^ cells/well) were transfected with 5 μg of one of the above described plasmids using calcium phosphate transfection.

### Recombinant GST-testin (variant) production and purification

The empty pGEX-2TK or pGEX-4T-2 vectors or those encoding a GST-testin variant were transformed in the *E*. *coli* BL21(DE3) pLYSstar strain. Bacteria were pre-cultured at 37°C for 4 h in Luria Bertani (LB) medium supplemented with 1% glucose. This pre-culture was diluted (1/25) in LB medium and grown at 37°C until an optical density of 0.6. Recombinant protein expression was induced by isopropyl-β-D-thiogalactopyranoside (1 mM) in LB medium that was supplemented with 10 μM zinc-acetate and grown at 16°C overnight. Bacterial cells were lysed in 25 mM Hepes (pH 7.6), 0.1 mM EDTA, 5 mM dithiothreitol, complete protease inhibitor cocktail (Roche) and 10.000 units/ml lysozyme (Amersham) and sonicated for 5 min on ice. GST or the GST-fusion protein was purified from the clarified lysate (from a 25 ml cell culture) by incubation for 2 h at 4°C with 25 μl glutathione sepharose^™^ 4B (GE Healthcare) in buffer A (25 mM Hepes (pH 7.6), 150 mM NaCl, complete protease inhibitor cocktail). After extensive washing in buffer A, the resin was used in the binding assay. See also scheme Fig 2A.

### In vitro binding assay and detection

To cleave GST from a testin variant, the GST-testin variant coupled to glutathione resin was treated for 15 min at 22°C with 100 units/ml thrombin (Sigma) in buffer A with omission of protease inhibitors. The cleaved, non-tagged testin variant was enriched in the flow through and the co-eluting thrombin was inactivated by adding complete protease inhibitor cocktail and 1 mM benzamidine (Sigma). To analyse binding between two testin variants, 5 μg of a thus obtained non-tagged testin variant, used as prey, was incubated for 2 h at 4°C in buffer A with glutathione sepharose resin (25 μl) to which a specified GST-testin variant, used as bait. Beads loaded with GST were used as negative control (see also Fig 2A). After incubation, the beads were washed three times in buffer A at 4°C, resuspended in sample buffer (65 mM Tris HCl (pH 6.8), 5% SDS, 250 mM dithiothreitol, 20% glycerol and 0.2% bromophenol blue) and heated to 95°C for 5 min prior to SDS-PAGE analysis on 10% gels. The proteins retained on the resin (the GST-tagged bait and its possible binding partner) where detected by Coomassie staining or performing western blotting. For western blotting, after transfer, the membranes (Whatman Protran nitrocellulose (Sigma)) were blocked for 1 h at room temperature in Odyssey blocking buffer (Li-Cor) and incubated overnight at 4°C with a primary antibody in Odyssey blocking buffer. Membranes were washed three times in PBS, 0.1% Tween 20 (Sigma) and incubated for 1 h at room temperature with a secondary antibody in Odyssey blocking buffer. After 3 washes in PBS, 0.1% Tween 20 and one in MilliQ-H_2_O, signals were detected on an Odyssey infrared imaging device (Li-Cor Biosciences). The mouse anti-testin antibody (ab57292, Abcam), rabbit anti-GST antibody (ab9085, Abcam), rabbit anti-Evl ([35]) or rabbit anti-cofilin (ACLF02, Cytoskeleton) were used at a 1/1000 dilution. The secondary goat anti-mouse IRdye800 or goat anti-rabbit IRdye680 antibodies (Li-Cor Biosciences) were used at 1/10000.

### Size exclusion chromatography

Recombinant FL was produced and purified as described above and subsequently dialysed overnight at 4°C in buffer A. Untagged FL protein (1.5 mg or as specified) was loaded on a Superdex ^™^ 200 HR 10/30 (GE Healthcare) column in a volume of 200 μl and separated at a flow rate of 0.8 ml/min on an AKTA system (GE Healthcare). Fractions (500 μl) were collected and analysed by SDS-PAGE. Where needed, samples were concentrated using Microcon-10kDa Centrifugal Filter Units (Millipore). The column was calibrated under identical conditions using a mixture of five standard proteins (Amersham): chymotrypsin (25 kDa), ovalbumin (45 kDa), albumin (68 kDa), aldolase (158 kDa) and catalase (240 kDa).

### Supernatans depletion assay

The affinity of the PET^52-233^ -LIM1-3 interaction was determined according to the supernatans depletion assay as recommended by Pollard [21]. Both proteins were produced and purified as described above. We prepared a batch of GST-LIM1-3 on glutathione beads and diluted these so that the final concentration of GST-LIM1-3 was 0,31; 0,63; 1,25; 2,50; 5; 10; 20; 30; 40 and 50 μM (the concentration indicated is based on the known amount of protein immobilized and the total volume of the incubation mixture). Each condition was incubated with 2.5 μM PET^52-233^ in buffer A for 1 hour at 4°C. In a negative control reaction, aspecific binding was assessed by incubation of the highest amount of glutathione-sepharose beads, without GST-LIM1-3, with a similar amount of PET^52-233^. Beads were spinned down by centrifugation. Identical volumes of the supernatans containing PET^52-233^ were analysed by SDS-PAGE followed by Coomassie staining. The unbound PET^52-233^ intensities were quantified using the Image Studio Lite software version 5.2 from Li-Cor and expressed as a % of the signal in the control sample (2.5 μM PET^52-233^ not incubated with beads, i.e. no binding, 100% unbound). Based hereupon, the % of bound PET^52-233^ was calculated for each condition and plotted versus the used GST-LIM1-3 concentrations. Curve fitting and subsequent calculation of the equilibrium dissociation constant (*K*_d_) was done in SigmaPlot13. We used the ‘one site saturation + nonspecific’ fitting setting because a considerable amount of aspecifical PET^52-233^ binding to beads was evident from the negative control reaction (2.5 μM PET^52-233^ incubated with beads lacking GST-LIM1-3). The fitting equation used in Sigmaplot is *y = (x)*.*B*_*max*_ */ (K*_*d*_ *+ (x)) + (x)*. *Ns* with x: the GST-LIM1-3 concentration, y: the bound fraction of PET^52-233^, B_max_: maximal PET^52-233^ binding, *K*_d_: equilibrium dissociation constant and N*s*: the portion of aspecific binding.

### Circular dichroism

Samples containg untagged testin variants were produced and purified as described above and dialysed overnight at 4°C in 10 mM NaH_2_PO_4_ pH 8. Measurements were performed with a scan resolution of 0,5 nm and 15 spectra were accumulated per sample using a J-710 Jasco spectropolarimeter. Buffer values were subtracted, curves were smoothened with the moving average method and the molar ellipticity was calculated using the J-710 Jasco software version 1.11.01.

### Immunofluorescent staining and microscopy

For microscopy experiments, the myc/mito tagged testin variants and GFP tagged testin variants were co-expressed in HeLa cells. Immunofluorescent staining was performed after fixation of the cells. The myc/mito tagged testin variants were detected using a primary mouse anti-c-myc antibody (9E10, Millipore), and a secondary Alexa Fluor^®^ 594 conjugated goat anti-mouse antibody (Invitrogen). Cells were imaged using a Zeiss LSM 510 Meta laser scanning confocal microscope (Carl Zeiss, Jena, Germany) with a Plan-Apochromat 63x/1.40 Oil DIC M27 objective. Images were processed using ZEN 2009 software. To monitor the subcellular localisation of FL-GFP and FL-Y288-GFP, constructs were overexpressed in HeLa cells, fixed and co-stained with a primary mouse anti-vinculin antibody (SIGMA) and a secondary Goat anti-mouse Alexa 594 antibody. Cells were visualised using an epifluorescence microscope (Leica DMRX, HCX PL APO 100x/1.35NA oil immersion lens).

### Alignments

Alignment of the LIM domains was performed using the tool at multalin (http://multalin.toulouse.inra.fr/multalin/).

## Supporting information

S1 Fig Size exclusion chromatography calibration curve and DTT treatment of dimeric testin fractions. A) SEC calibration curve (log molecular weight is plotted versus elution volume (ml)) obtained using five proteins of which molecular weight and elution volume are displayed in the chart. Red and green dots show the elution volume of monomeric and dimeric testin, respectively, and correspond to an estimated molecular weight of approximately 60 kDa and 98 kDa respectively. B) Coomassie stained SDS-PAGE analysis of collected dimeric testin fractions obtained after SEC analyses (see also Fig 3 top panel). Samples were treated either without (1–3) or with the reducing agent DTT (250 mM) (4–6). 5μg (1 and 4), 10μg (2 and 5) and 20 μg (3 and 6) of protein was loaded for analysis. Only a monomeric testin signal (~50 kDa) was detected for the non-treated conditions (1–3) indicating the absence of a dimeric oxidation product. M: marker proteins (kDa).
(TIF)

S2 Fig The PET domain of testin is not sufficient for interaction with LIM1-3 domains *in vitro*. The experimental setup is similar as the scheme in Figs 2A and 4. Immobilised GST-LIM1-3 on glutathione resin was incubated with untagged PET^52-233^(A), PET^92-199^(B), PET^52-199^ (C) or PET^92-233^ (D) in solution, used as preys. Coomassie stained SDS-PAGE analysis is shown: input (I) shows the untagged prey protein prior to incubation with the resin. Lanes indicated with P1 show the proteins present on the resin. An extra negative control was incorporated: the immobilised baits on the resin were mock-incubated with a solution lacking the soluble preys because the used bait constructs is sometimes prone to degradation during immobilization on the resin (lanes labelled P2). GST-cofilin resin was used as second negative control in each setup (lanes P3). Untagged protein bound to the GST-testin variant immobilised on the resin is highlighted by a red box (A). Positions of bait, prey and negative control (Neg Ctr) bait are indicated in each panel, M: marker proteins (kDa).
(TIF)

S3 Fig Circular dichroism of PET^52-233^ and PET^92-199^. CD spectra of PET^52-233^ (blue) and PET^92-199^ (red). Molar ellipticity [deg cm^2^ dmol-1] is plotted versus wavelength (nm). Spectra of PET^52-233^ and PET^92-199^ evidence these fragments of testin are structured.
(TIF)

S4 Fig Interaction between PET^52-233^ and LIM1-2 in cells requires both LIM1 and LIM2 domains. Immunofluorescent staining of myc/mito-tagged (bait) and GFP-tagged (prey) testin constructs in HeLa cells. Myc signal (red), GFP signal (green) are shown for each condition. PET^52-233^ myc/mito was used as bait (A-F). Note the dotted appearance of the (red) bait signal consistent with targeting to the mitochondria via the mito-tag. When used as prey, LIM1-2-GFP was recruited by PET^52-233^(A, see also Fig 6C). No recruitment is observed when LIM2-3-GFP, LIMΔ2-GFP, LIM1-GFP, LIM2-GFP or LIM3-GFP were used as prey (B-F).
(TIF)
