# Supplementary figures and images for: The PET and LIM1-2 domains of testin contribute to intramolecular and homodimeric interactions
Source: PLoS One. 2017 May 18;12(5):e0177879. doi: 10.1371/journal.pone.0177879 (PMC5436826; doi:10.1371/journal.pone.0177879)

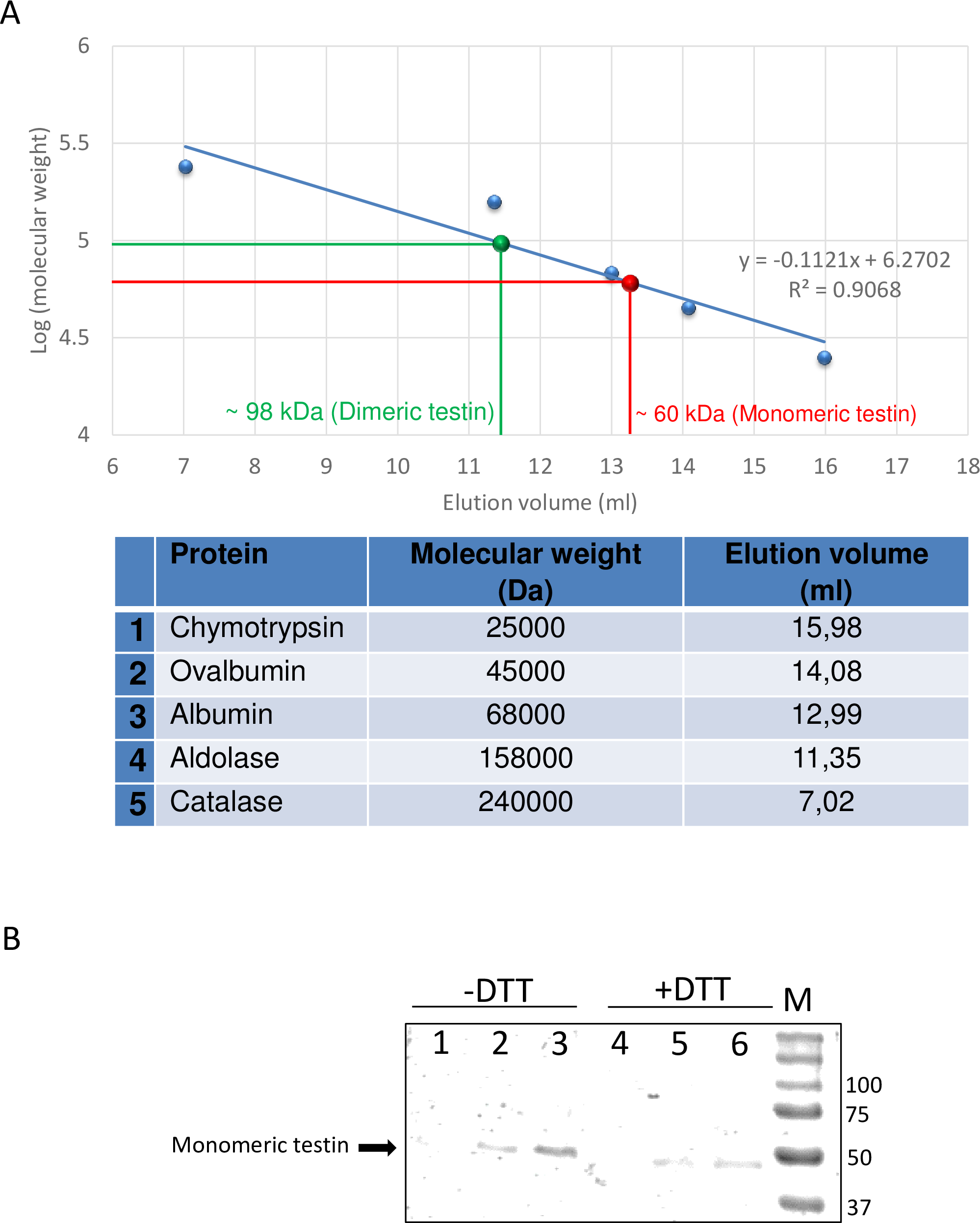

Supplement: S1 Fig — A) SEC calibration curve (log molecular weight is plotted versus elution volume (ml)) obtained using five proteins of which molecular weight and elution volume are displayed in the chart. Red and green dots show the elution volume of monomeric and dimeric testin, respectively, and correspond to an estimated molecular weight of approximately 60 kDa and 98 kDa respectively. B) Coomassie stained SDS-PAGE analysis of collected dimeric testin fractions obtained after SEC analyses (see also Fig 3 top panel). Samples were treated either without (1–3) or with the reducing agent DTT (250 mM) (4–6). 5μg (1 and 4), 10μg (2 and 5) and 20 μg (3 and 6) of protein was loaded for analysis. Only a monomeric testin signal (~50 kDa) was detected for the non-treated conditions (1–3) indicating the absence of a dimeric oxidation product. M: marker proteins (kDa). (TIF) [file pone.0177879.s001.tif]

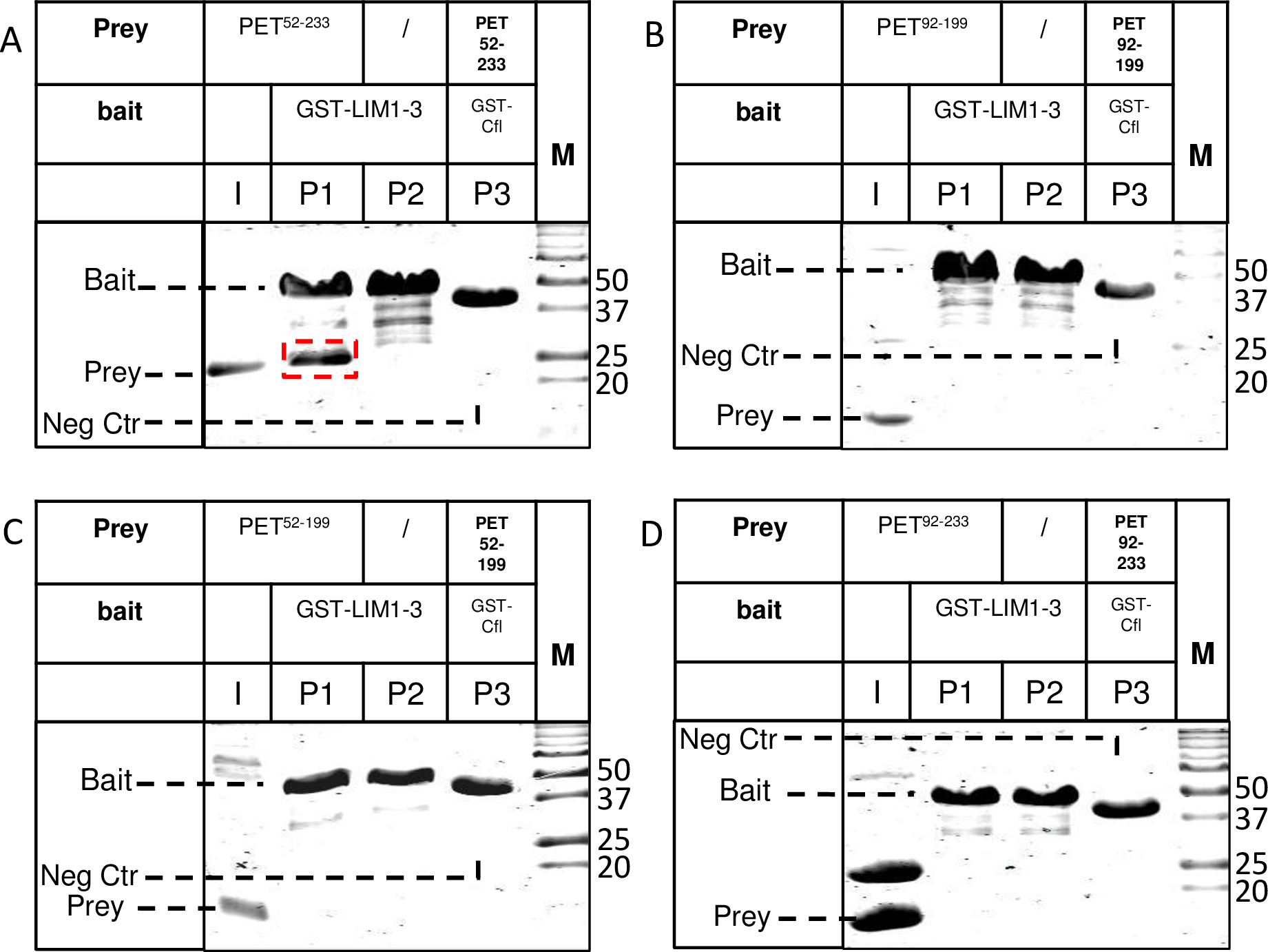

Supplement: S2 Fig — The experimental setup is similar as the scheme in Figs 2A and 4. Immobilised GST-LIM1-3 on glutathione resin was incubated with untagged PET52-233(A), PET92-199(B), PET52-199 (C) or PET92-233 (D) in solution, used as preys. Coomassie stained SDS-PAGE analysis is shown: input (I) shows the untagged prey protein prior to incubation with the resin. Lanes indicated with P1 show the proteins present on the resin. An extra negative control was incorporated: the immobilised baits on the resin were mock-incubated with a solution lacking the soluble preys because the used bait constructs is sometimes prone to degradation during immobilization on the resin (lanes labelled P2). GST-cofilin resin was used as second negative control in each setup (lanes P3). Untagged protein bound to the GST-testin variant immobilised on the resin is highlighted by a red box (A). Positions of bait, prey and negative control (Neg Ctr) bait are indicated in each panel, M: marker proteins (kDa). (TIF) [file pone.0177879.s002.tif]

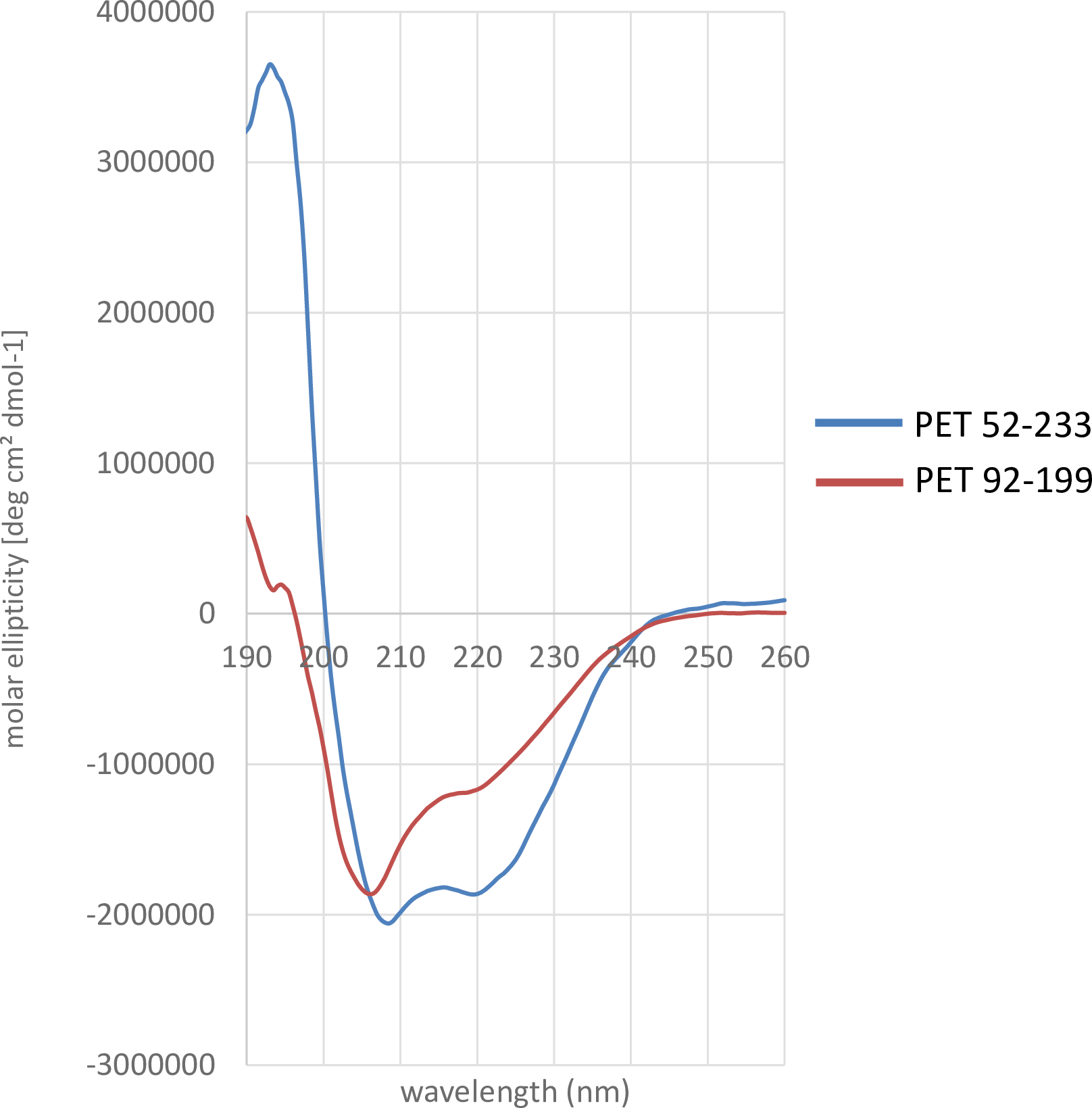

Supplement: S3 Fig — CD spectra of PET52-233 (blue) and PET92-199 (red). Molar ellipticity [deg cm2 dmol-1] is plotted versus wavelength (nm). Spectra of PET52-233 and PET92-199 evidence these fragments of testin are structured. (TIF) [file pone.0177879.s003.tif]

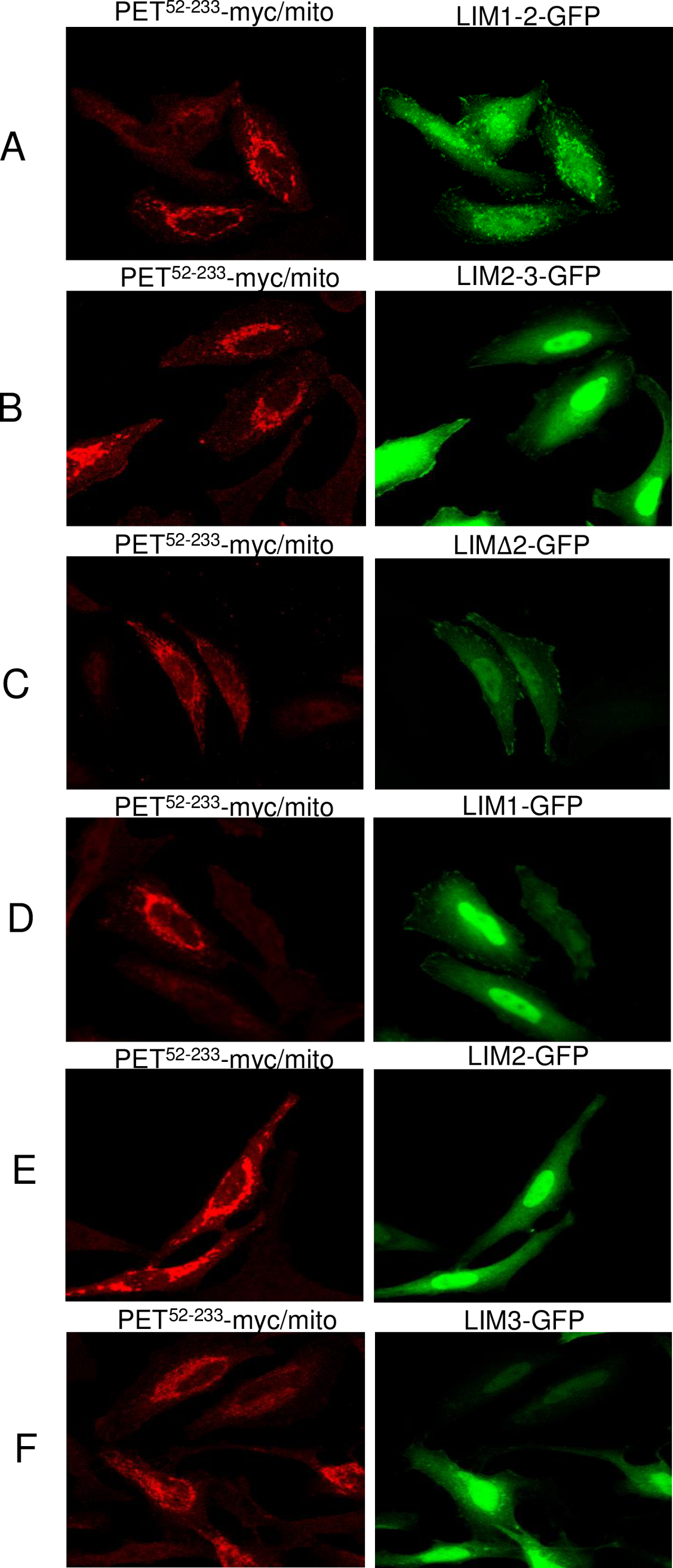

Supplement: S4 Fig — Immunofluorescent staining of myc/mito-tagged (bait) and GFP-tagged (prey) testin constructs in HeLa cells. Myc signal (red), GFP signal (green) are shown for each condition. PET52-233 myc/mito was used as bait (A-F). Note the dotted appearance of the (red) bait signal consistent with targeting to the mitochondria via the mito-tag. When used as prey, LIM1-2-GFP was recruited by PET52-233(A, see also Fig 6C). No recruitment is observed when LIM2-3-GFP, LIMΔ2-GFP, LIM1-GFP, LIM2-GFP or LIM3-GFP were used as prey (B-F). (TIF) [file pone.0177879.s004.tif]
